# Current News

**Published:** 1889-10

**Authors:** 


### Current News.

---

HAVE you "seen" Dr. Crouse yet?

It will be necessary for you to do so if you want his assistance when that injunction comes for those crowns and bridges you have made.

Remember that in organization there is strength, and surely there never was a time when the profession needed to meet organization with organization as at the present.

The value of such a movement cannot be estimated. Its effects will be far-reaching. Let us stand by each other in the time of need, and success will surely crown our efforts.

---

WE still continue the *special offer* of the November and December numbers free to all new subscribers who send in their names for 1890 before November 15, 1889.

---

ACCEPTING the "chemical theory" of caries, Dr. G. W. McElhaney traces the original cause back to the acid in the primeval apple eaten by Adam. Accepting the "germ theory," Dr. W. C. Browne finds it in the bugs and worms in the same apple.

---

WITH the bur in the engine the difference between live and dead bone is like cutting cheese and striking the rind. So says Dr. S. A. White.

---

THE "germ theory," at first ridiculed as the "bug" theory, the "worm" theory, etc., has come to stay, and in my mind fulfils all the requirements of a perfect theory and explains the hitherto obscure phenomena of caries more satisfactorily than anything which preceded it. W. C. WARDLAW.

---

A PIECE of zinc placed in the vulcanizer prevents the flasks from soiling the hands. W. C. BROWNE.

UNDER the title "Man and his World," the J. B. Lippincott Co. is to publish in a few weeks the matter of the philosophical lectures delivered last winter by Dr. Garretson. Gutekunst is preparing a fine photograph as a frontispiece for this volume.

---

### UNION DENTAL CONVENTION.

THE twenty-first Annual Union Convention of the Fifth, Sixth, Seventh, and Eighth District Dental Societies of the State of New York, will be held in Stancliff Hall, Elmira, N. Y., Tuesday and Wednesday, October 29 and 30, 1889. A programme of interest to practical every-day dentists has been secured, and the committee feel assured that every dentist within the borders of the several districts represented will feel a personal interest in the meeting. A large attendance is already assured. Persons desiring to make exhibits should apply to Dr. F. B. Darby, Elmira, N. Y., chairman of the Committee of Arrangements.

MYRON D. JEWELL,
Richfield Springs, N. Y.,
*Chairman Business Committee.*

---

### UNION DENTAL MEETING.

THE Connecticut Valley Dental Society, New England Dental Society, and the Connecticut State Dental Society will hold a union meeting at Springfield, Mass., on Wednesday, Thursday, and Friday, October 23, 24, and 25. Lectures, essays, clinics, and dental technics will make up the programme. All members of dental societies are cordially invited. Reduced railroad rates will be secured. Programmes containing all particulars of the meeting will be issued by October 15, and will be sent to any address by applying to

GEO. A. MAXFIELD, D.D.S.,
*Secretary Connecticut Valley Dental Society,*
*Holyoke, Mass.*

EDGAR O. KINGMAN, D.D.S.,
*Secretary New England Dental Society,*
*Cambridge, Mass.*

GEO. L. PARMELE, M.D.,
*Secretary Connecticut State Dental Society,*
*Hartford, Conn.*

